# Stabilized coronavirus spikes are resistant to conformational changes induced by receptor recognition or proteolysis

**DOI:** 10.1038/s41598-018-34171-7

**Published:** 2018-10-24

**Authors:** Robert N. Kirchdoerfer, Nianshuang Wang, Jesper Pallesen, Daniel Wrapp, Hannah L. Turner, Christopher A. Cottrell, Kizzmekia S. Corbett, Barney S. Graham, Jason S. McLellan, Andrew B. Ward

**Affiliations:** 1 Department of Integrative Structural and Computational Biology, The Scripps Research Institute, La Jolla, CA 92037 USA; 2 Department of Biochemistry and Cellular Biology, Geisel School of Medicine at Dartmouth, Hanover, NH 03755 USA; 3 Present Address: Department of Molecular Biosciences, The University of Texas at Austin, Austin, TX 78712 USA; 4 Vaccine Research Center, National Institute of Allergy and Infectious Diseases, Bethesda, MD 20814 USA

## Abstract

Severe acute respiratory syndrome coronavirus (SARS-CoV) emerged in 2002 as a highly transmissible pathogenic human betacoronavirus. The viral spike glycoprotein (S) utilizes angiotensin-converting enzyme 2 (ACE2) as a host protein receptor and mediates fusion of the viral and host membranes, making S essential to viral entry into host cells and host species tropism. As SARS-CoV enters host cells, the viral S is believed to undergo a number of conformational transitions as it is cleaved by host proteases and binds to host receptors. We recently developed stabilizing mutations for coronavirus spikes that prevent the transition from the pre-fusion to post-fusion states. Here, we present cryo-EM analyses of a stabilized trimeric SARS-CoV S, as well as the trypsin-cleaved, stabilized S, and its interactions with ACE2. Neither binding to ACE2 nor cleavage by trypsin at the S1/S2 cleavage site impart large conformational changes within stabilized SARS-CoV S or expose the secondary cleavage site, S2′.

## Introduction

Severe acute respiratory syndrome coronavirus (SARS-CoV) emerged in humans from an animal reservoir in 2002 and rapidly spread globally causing 8,096 cases and 774 associated deaths in 26 countries through July 2003^1^. SARS-CoV reappeared in a second smaller outbreak in 2004, but has since disappeared from human circulation. However, closely related coronaviruses, such as WIV1, currently circulate in bat reservoirs and are capable of utilizing human receptors to enter cells^2^ and there are no vaccines or virus-specific treatments available for human use. The more recent emergence of Middle East respiratory syndrome coronavirus (MERS-CoV)^1^ and the likelihood of future zoonotic transmission of novel coronaviruses to humans from animal reservoirs makes robust reagent development for the display of neutralizing epitopes of great importance to human health. Understanding how coronavirus S glycoproteins are processed and bind to host receptors is key to the development of coronavirus vaccines and therapeutics.

Coronaviruses are enveloped viruses possessing large, trimeric spike glycoproteins (S) required for the recognition of host receptors for many coronaviruses as well as the fusion of viral and host cell membranes for viral entry into cells^3^. During viral egress from infected host cells, some coronavirus S proteins are cleaved into S1 and S2 subunits. The S1 subunit is responsible for host-receptor binding while the S2 subunit contains the membrane-fusion machinery. During viral entry, the S1 subunit binds host receptors in an interaction thought to expose a secondary cleavage site within S2 (S2′) adjacent to the fusion peptide for cleavage by host proteases^4–7^. This S2′ proteolysis has been hypothesized to facilitate insertion of the fusion peptide into host membranes after the first heptad repeat region (HR1) of the S2 subunit rearranges into an extended α-helix^8–10^. Subsequent conformational changes in the second heptad repeat region (HR2) of S2 form a six-helix bundle with HR1, fusing the viral and host membranes and allowing for release of the viral genome into host cells^10^. Coronavirus S is also the target of neutralizing antibodies^11^, making an understanding of S structure and conformational states pertinent for investigating S antigenic surfaces and designing vaccines.

The SARS-CoV S1 subunit is composed of two distinct domains: an N-terminal domain (S1 NTD) and a receptor-binding domain (S1 RBD) also referred to as the S1 CTD or domain B. Each of these domains have been implicated in binding to host receptors, depending on the coronavirus in question. However, most coronaviruses are not known to utilize both the S1 NTD and S1 RBD for viral entry^12^. SARS-CoV makes use of its S1 RBD to bind to the human angiotensin-converting enzyme 2 (ACE2) as its host receptor^13,14^.

Recent examination using cryo-electron microscopy (cryo-EM) has illuminated the prefusion structures of coronavirus spikes^15–22^. Initial examination of HCoV-HKU1 S showed that the receptor-binding site on the S1 RBD was occluded when the RBD was in a ‘down’ conformation and it was hypothesized that conformational changes were required to access this site^16^. Subsequent studies of the highly pathogenic human coronavirus S proteins of SARS-CoV^15,22^ and MERS-CoV^17,22^ showed that these viral S1 RBD do indeed sample an ‘up’ conformation where the receptor-binding site is accessible. These structural studies also located the positions of the S1/S2 and S2′ cleavage sites on the prefusion spike. The S1/S2 site lies within a surface exposed loop in the second subdomain of S1^16^. However, the S2′ site lies closer to base of the spike and though this region is located on the surface of the spike, cleavage at this site is prevented by surrounding protein elements^17^.

Coronavirus S transitions from a meta-stable prefusion state to a highly stable postfusion state as part of the S protein’s role in membrane fusion. The instability of the prefusion state presents a significant challenge for the production of protein antigens for antigenic presentation of the prefusion antibody epitopes that are most likely to lead to neutralizing responses. Recently, we presented the design of two proline mutations (2P) for the prefusion stabilization of coronavirus S proteins^17^. The stabilized MERS-CoV S 2P ectodomain was shown to maintain the prefusion spike conformation, have similar antibody recognition as wild-type S and possess higher immunogenicity. Here, we have used single-particle cryo-EM to determine structures of prefusion stabilized SARS-CoV S 2P ectodomain in uncleaved, S1/S2 cleaved and ACE2-bound states. Three-dimensional classification of the S1 RBD positions and corresponding atomic protein models revealed that neither ACE2-binding nor trypsin cleavage at the S1/S2 boundary induced substantial conformational changes in the prefusion stabilized S protein.

## Results

### Structural description of SARS-CoV S 2P ectodomain

**Figure 1:**
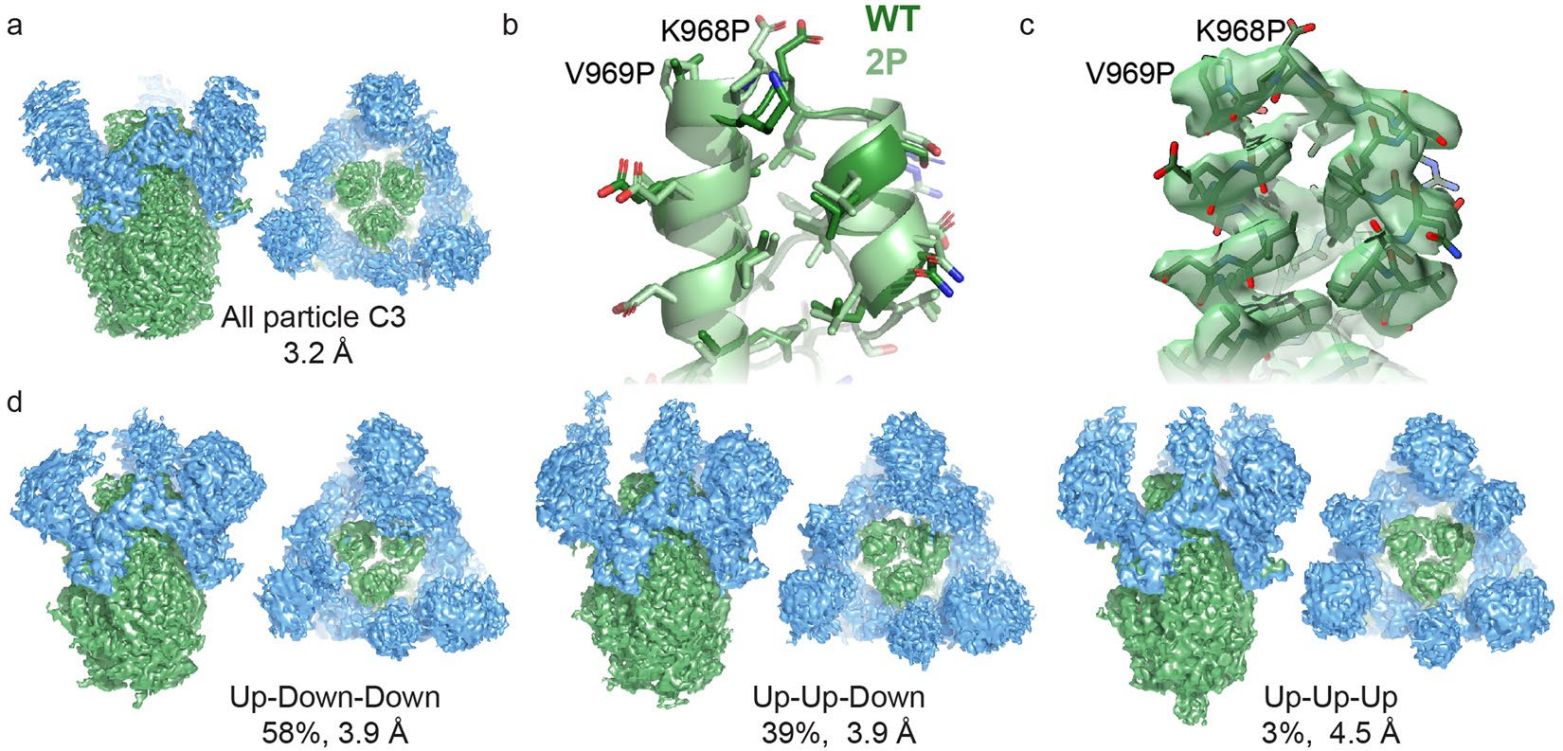
Structure of the SARS-CoV S 2P ectotodomain. (**a**) The C3 symmetrized reconstruction of all particles within the dataset resembles that of other betacoronaviruses. Side and membrane-distal top views (90° rotation about x-axis) are shown. (**b**) Coordinate models derived from cryo-EM reconstructions of the wild-type SARS-CoV S ectodomain^22^ (5X58.pdb, dark green) and the prefusion stabilized SARS-CoV S 2P ectodomain (6CRV.pdb light green) adopt identical conformations near the 2P mutation site. Coordinate models derived from C3 symmetry cryo-EM reconstructions are shown. (**c**) There is highly featured density in the region containing the 2P mutation site. (**d**) Classification of heterogeneity within the S1 RBD reveals a distribution of RBD configurations with the single-‘up’ conformation being most prevalent. S1 regions are shown in blue and S2 regions are shown in green.

To examine any potential conformational differences in SARS-CoV S 2P compared to unstabilized wild-type S ectodomains^15,22^, we determined structures of the SARS-CoV S 2P ectodomain. This included a 3.2 Å resolution C3-symmetric reconstruction and several asymmetric reconstructions (3.9–4.5 Å, see below) (Fig. 1 and Supplementary Tables S1, S4 and Figs S1, S2). The S1 NTD and some of the S1 RBD, while clearly present, appear poorly resolved in the cryo-EM maps, precluding a *de novo* build of these regions. For this reason, available SARS-CoV S crystal structures^22,23^ of corresponding domains were placed into these densities. Overall, the SARS-CoV S 2P structure resembles that of other coronaviruses, particularly those of the betacoronavirus genus^16,17,19,22^ and the previously published SARS-CoV wild-type prefusion ectodomains^15,22^. The S1 domains surround the helical S2 subunits and interdigitate at the membrane-distal apex of the trimeric spike. The structure of the S2 subunit appears to be highly conserved across coronavirus genera^18,20,21^. The SARS-CoV prefusion wild-type and 2P structures are nearly identical in most regions, including regions proximal to the 2P mutation site (Fig. 1b,c).

**Figure 2:**
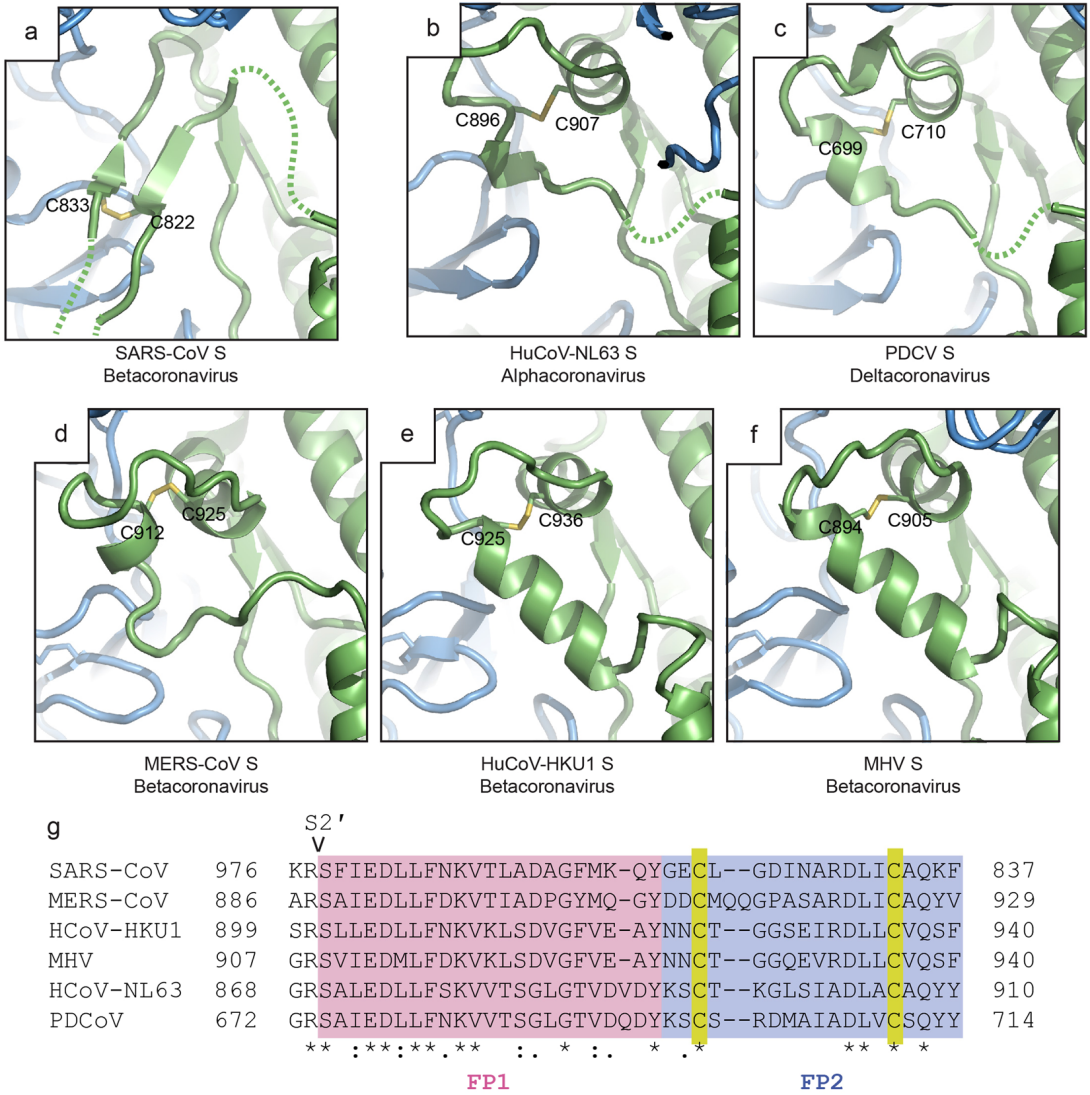
Comparison of putative bi-partite fusion peptide regions. (**a**) The SARS-CoV S FP2 region of the bi-partite fusion peptide^24^ adopts a conformation distinct from equivalent regions in (**b**) alpha- (HuCoV-NL63, 5SZS.pdb^20^) and (**c**) deltacoronavirus spikes (PDCV S, 6BFU.pdb^21^) as well as other (**d**–**f**) betacoronaviruses (MERS-CoV S, 5W9I.pdb^17^, HuCoV-HKU1 S, 5I08.pdb^16^, MHV S, 3JCL.pdb^19^). Subunits are colored as in Fig. 1. (**g**) Sequence alignment of the fusion peptide regions of coronavirus spikes highlighting the FP1 and FP2 regions as well as the cysteine residues involved in disulfide bond formation indicated in panels a–f. Sequence alignment was prepared with Clustal Omega^52^.

Although the overall structures of SARS-CoV S are similar, we were able to build several S2 protein regions excluded from the wild-type SARS-CoV S models, including a fusion-peptide-adjacent region as well as additional amino acids in the S2 C-terminal connector domain. The region C-terminal to the fusion peptide, known as FP2, has been suggested to act as the second part of a bipartite fusion peptide for SARS-CoV S^24^. Comparison of this FP2 region in the structures presented here with the MHV^19^, HCoV-HKU1^16^, MERS-CoV^17,22^, HCoV-NL63^20^ and Porcine deltacoronavirus^18,21^ S structures indicates that SARS-CoV adopts a unique conformation in this region (Fig. 2). Whereas most other coronaviruses possess two short α-helices with a cross-linking disulfide bond, in SARS-CoV a similar disulfide bond bridges the two strands of a beta-hairpin. The unique SARS-CoV S conformation of FP2 and the lack of cross-group sequence conservation beyond the disulfide bond in this region suggest that SARS-CoV may use a distinct mechanism of FP2 membrane insertion.

The trimeric SARS-CoV S 2P adopts two distinct conformations related to each of the S1 RBD: ‘up’ and ‘down’. The delineation of two distinct conformations for each S1 RBD is consistent with observations made for MERS-CoV^17,22^ as well as one of the studies of wild-type SARS-CoV S^22^. Gui *et al*.^15^, however, observed a gradient of conformations in wild-type SARS-CoV S, which has not been observed in other structural studies of coronavirus spikes nor the work presented here. Some possibilities for these different observations may be the differences in spike expression system yielding alternate glycosylation patterns or alternate protein constructs, but the true source remains unclear. The ‘down’ conformation caps the S2 helices and makes extensive contacts with the S1 NTD. The ‘up’ conformation of the S1 RBD exposes the S1 RBD receptor-binding site. It has been previously reported that for wild-type SARS-CoV S, 56% of the particles contained three ‘down’ RBD conformations while 44% contained a single ‘up’ S1 RBD conformation^22^. To examine the conformation of the S1 RBD among our SARS-CoV S 2P ectodomains, we used a local masking, image sutraction and 3-D sorting strategy^17^ to more accurately classify the conformations as being either ‘down’ or ‘up’ at each of the three S1 RBD positions within the trimer. This analysis revealed that the majority of the S 2P proteins were in the single-‘up’ conformation (58%) with lesser amounts of double- and triple-‘up’ conformations (39% and 3% respectively) and with no all-‘down’ conformation observed. The increased propensity to adopt the ‘up’ S1 RBD conformation may indicate a difference in the coronavirus S containing the 2P mutations, however other differences in sample preparation cannot be fully ruled out.

### ACE2 and S1 C-terminal domains

**Figure 3:**
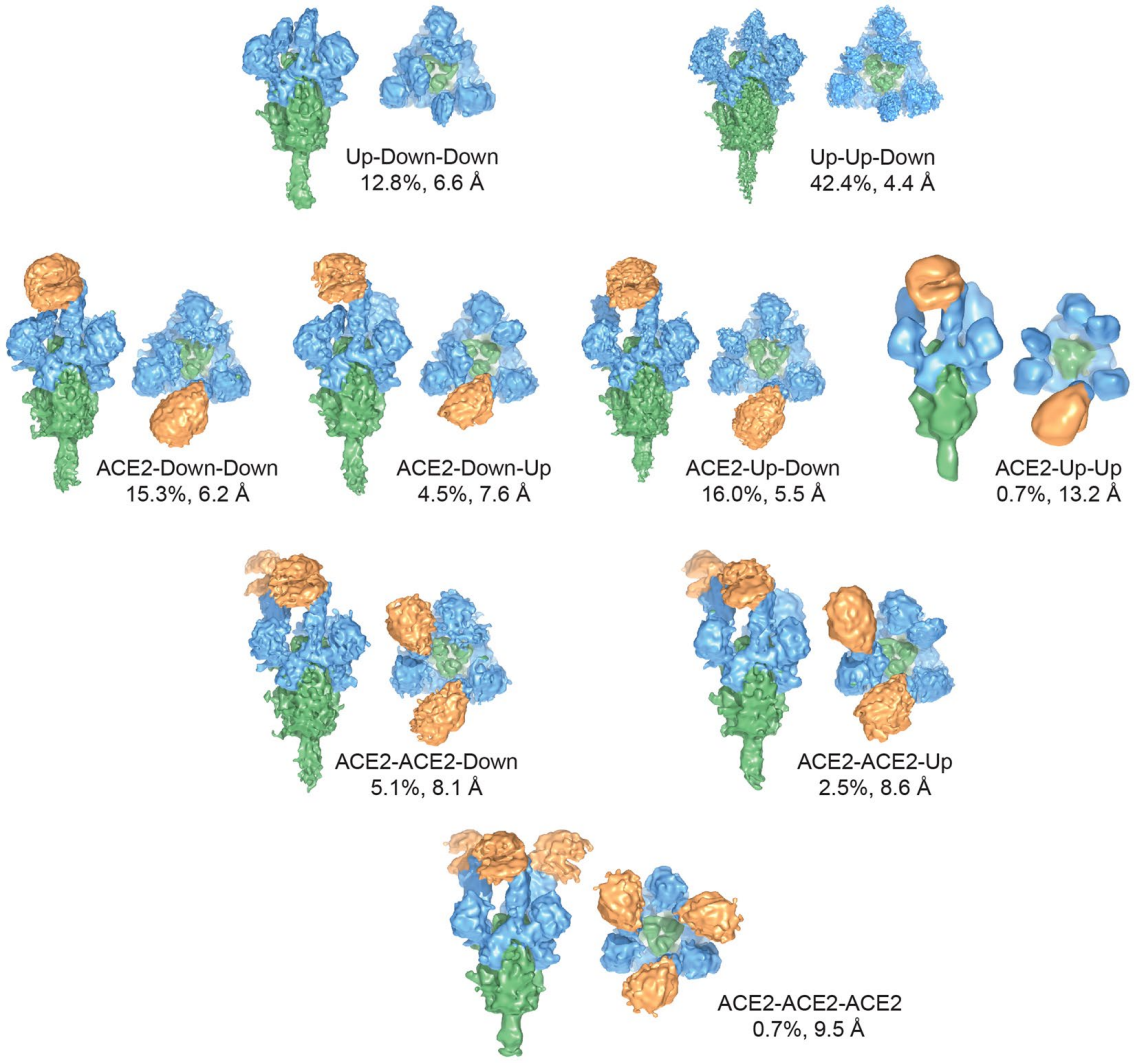
Compositional and conformational heterogeneity of ACE2-bound SARS-CoV spikes. Classification of heterogeneity within the SARS-CoV S – ACE2 complex reveals that 55% of the particles are not bound by ACE2. Of those particles which are bound by ACE2, each configuration of the S1 RBD is observed with classes containing triple ‘up’ conformation S1 RBD being the most poorly represented. The labeled description of each class begins at the lower RBD when viewed at the membrane distal apex and proceeds clockwise. Side views and membrane-distal top views are shown for each reconstruction. S1 regions are shown in blue, S2 regions are shown in green and soluble ACE2 is shown in orange.

To examine the structural consequences binding ACE2 to the stabilized SARS-CoV S 2P, we combined SARS-CoV S 2P ectodomain with an excess of soluble human ACE2 with subsequent purification by size-exclusion chromatography and immediate cryo-EM specimen preparation (Supplementary Fig. S1). Initial sorting of particle heterogeneity indicated spikes could be split into ACE2-bound (45%) and unbound (55%) classes. Using a similar masking and 3-D sorting strategy as above we sorted the unbound S class further into classes with S1 conformations of one or two ‘up’ S1 RBDs (Fig. 3 and Supplementary Tables S2–S4 and Figs S3–S5). We did not observe an all-‘down’ class nor a three ‘up’ S1 RBD class indicating a low prevalence of these conformations among the unbound S 2P proteins. Expanding our 3D sorting strategy, we classified our ACE2-bound particles at each S1 RBD position and identified single, double and triple ACE2-bound S. We were further able to identify S1 RBD conformations at the non-ACE2 occupied RBD positions to represent each population of S1 RBD conformations among ACE2-bound S.

As hypothesized by previous structural work^15–17,22^, the S1 RBD of the stabilized spike recognizes ACE2 with an ‘up’ S1 RBD conformation. The proportion of total ‘up’ S1 RBD conformations within the ACE2-bound and -unbound classes is nearly identical within this dataset (58% ‘up’ S1 RBD). This strongly suggests that binding of a single ACE2 receptor does not induce adjacent S1 RBDs to transition from a ‘down’ to ‘up’ conformation within the context of the stabilized S. Hence, ACE2 is more likely to bind to an already ‘up’ S1 RBD rather than inducing the conformational changes that are required for the S1 RBD to become accessible to ACE2. Though the proportion of ‘up’ S1 RBD in the ACE2 dataset (58%) is similar to the proportion of ‘up’ S1 RBD in the SARS S 2P ectodomain dataset (48%), there is a large difference in the distributions of one ‘up’ and two ‘up’ conformational states between these datasets. The significance of this difference remains unclear.

Despite prolonged co-incubation and an excess of ACE2, we had difficulties in saturating the S1 RBD with ACE2 in the context of stabilized trimeric S ectodomain. This poor saturation is illustrated by the small proportion of triple-bound ACE2 and the majority of spikes that are unbound by receptor. In contrast, isolated recombinant S1 RBD easily binds ACE2 and is capable saturating ACE2 on target cells to block S-mediated entry^14^. Our observed sub-stoichiometric ACE2 binding to trimeric spikes is consistent with the difficulty in using soluble ACE2 receptor to neutralize SARS-CoV S pseudotyped onto VSV^25^. The reduced binding of ACE2 to trimeric spikes is likely due to the incomplete exposure and conformational flexibility of the S1 RBD. In contrast, MHV which binds CEACAM1a via its S1 NTD, but does not undergo conformational changes exhibits complete neutralization by soluble receptor^19,26^.

**Figure 4:**
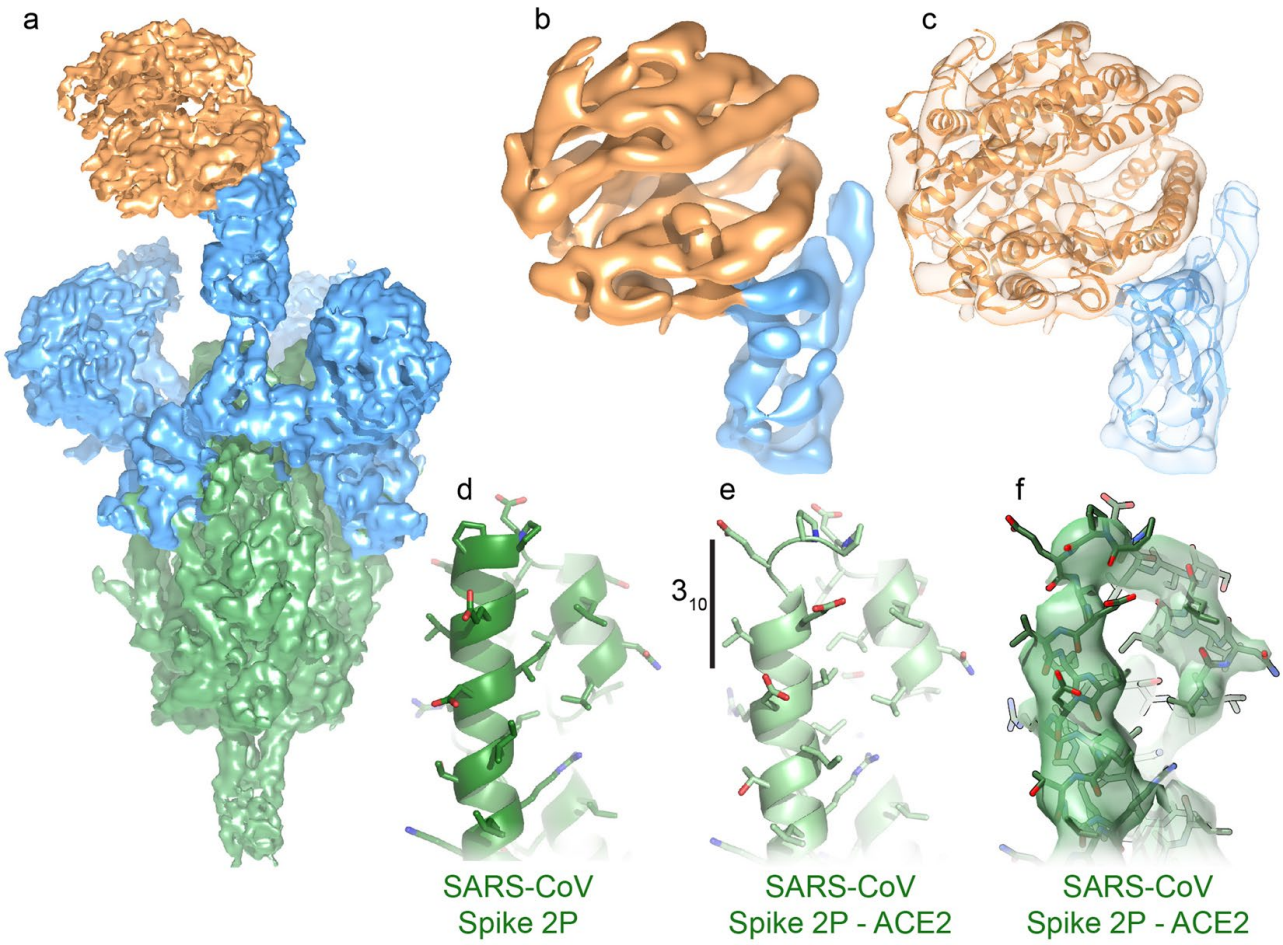
ACE2-receptor binding and induced conformational changes. (**a**) ACE2 binds the SARS-CoV S1 RBD in an ‘up’ conformation. (**b**) Focused refinement was used on the S1 RBD – ACE2 portion of the particles to improve the density of this flexible region. (**c**) Rigid-body fitting of the SARS-CoV S1 RBD – ACE2 complex crystal structure (2AJF.pdb^23^) indicates that the crystal structure is representative of the ACE2-bound trimeric spike. Comparison of the d) SARS-CoV S 2P central helices with the (**e**) SARS-CoV S 2P central helix which has been uncapped by an ACE2-bound S1 RBD demonstrates a transition to a short 3_10_-helix. (**f**) The ACE2-bound SARS-CoV S 2P central helix demonstrates good density in this region. Colors are as in Fig. 3.

Similar to recently published MERS-CoV S structures^17^, the ACE2-bound S1 RBD of SARS-CoV S 2P adopts a much more extended and rotated conformation compared to S1 RBD modeled in previous wild-type SARS-CoV S structures^22^ (Supplementary Fig. S6). This difference is likely due to poor density in the hinge regions between the S1 RBD and subdomain 1 (SD-1) in these previous reconstructions^15,22^ rather than the presentation of a unique receptor-bound conformation or the influence of the stabilizing mutations. Indeed, the bound ACE2 receptor and S1 RBD for all reconstructions here show poorer density quality than the less mobile regions of the SARS-CoV S (Fig. 4). To improve the density for ACE2-bound S1 RBD, we used focused refinement with image subtraction on this region to overcome the flexibility of these domains relative to the rest of SARS-CoV S 2P. This yielded a 7.9 Å resolution reconstruction with improved local density quality (Fig. 4b,c). We successfully placed the crystal structure of the SARS-CoV S1 RBD bound to ACE2 (2AJF.pdb^23^) into this density as a rigid body indicating that the previously determined crystal structure accurately recapitulates the conformation between the ACE2-bound S1 RBD in the prefusion stabilized trimeric spike. Given that the S1 RBD in its ‘up’ conformation is well separated in space and sequence from the 2P stabilizing mutations, we do not anticipate that the introduction of these prefusion stabilizing mutations influences the molecular interactions between the S1 RBD and ACE2 beyond perhaps increasing the prevalence of the S1 RBD ‘up’ conformations as noted above. Indeed, both the wild-type and 2P-stabilized SARS-CoV S ectodomains demonstrate similar affinities for ACE2 by SPR (185 and 150 nM respectively, Supplementary Fig. S7).

The ACE2-bound, S1 RBD extends upwards and rotates away from contacts with nearby amino acids. Hence, any conformational changes induced by receptor binding to the S1 RBD are more likely to be caused by the absence of the S1 RBD contacts in the ‘up’ conformation, rather than the formation of additional intra-spike contacts in a receptor-bound state (Supplementary Fig. S8). Indeed, movement of the S1 RBD to the ‘up’ conformation disrupts molecular interactions with the S2 central α-helices which may play a role in receptor-induced conformational changes in wild-type spikes. The observation that host-receptor engagement of the S1 RBD disrupts interactions within spike in a manner independent of direct receptor-spike contacts suggests a flexible mechanism for how different coronavirus spikes may bind to diverse protein receptors with their S1 RBD and facilitate fusion with host cells.

### SARS-CoV S2 fusion machinery and receptor binding

Several biochemical and virological studies have suggested that binding of a host protein receptor to coronavirus spikes induces conformational changes leading to fusion and exposure of the S2′ protease site for cleavage^4,6^. Comparison of our ACE2-bound 2P-stabilized spike structures with those of both the wild-type SARS-CoV S and the 2P-stabilized SARS-CoV S indicate that overall, these structures are similar and do not exhibit large conformational differences. The absence of conformational changes includes regions near the S2′ cleavage site, fusion peptide and S2 heptad repeat 1 (HR1). However, fine examination of the ACE2-bound S structure revealed a modest conformational change in the S2 central helix where the S2 had been uncapped by a receptor-bound RBD. In both the wild-type^15,22^ and 2P unbound structures, the central helix presents as an α-helix in each protomer of the trimer. However, in the ACE2-bound structure, the upper portion of the S2 helix uncapped by the ACE2-bound S1 RBD has transitioned to a 3_10_-helix (Fig. 4d,e). The α- to 3_10_-helix transition is unique to the S2 protomer uncapped by the ACE2-bound S1 RBD and is not observed in the other two copies of the trimeric spike. This subtle transition may indicate that conformational changes towards fusion are initiated in the central helix. N-terminal to the central helix are the 2P mutations that stabilize the spike in its prefusion conformation. These stabilizing mutations may block the propagation of conformational changes beyond the subtle change to a short 3_10_-helix, providing a possible mechanism for their prefusion stabilizing effect.

### Trypsin-cleaved SARS-CoV spike

Unlike some other coronavirus spikes such as MERS-CoV S, SARS-CoV S lacks an amino acid sequence capable of recognition by host furin proteases at the S1/S2 cleavage site. However, SARS-CoV S can be cleaved *in vitro* by exogenous trypsin at an equivalent site, and it has been proposed that a similar cleavage event may occur *in vivo* by trypsin-like proteases during viral entry^27,28^. Moreover, trypsin cleavage of the SARS-CoV S potentiates S for more efficient membrane fusion^27,29^. For MERS-CoV S, cleavage at S1/S2 enables more efficient receptor recognition^6^. In addition, coronaviruses possess a secondary cleavage site in S2 called S2′^27^. This site is thought to be cleaved by host proteases after receptor recognition^4–6^ and liberates the viral fusion peptide to insert into host membranes in a S2 pre-hairpin intermediate^30^. The S2′ cleavage has been proposed to occur after an arginine residue conserved across coronavirus genera (SARS-CoV S Arg797)^31^.

To examine the trypsin protease sensitivity of our prefusion-stabilized coronavirus spikes, we carried out limited proteolysis experiments of both wild-type and 2P SARS-CoV S ectodomains in the presence and absence of soluble ACE2 receptor. A time course of the proteolysis revealed that all four samples are cleaved to S1 and S2 products at equivalent rates at similar sites (Supplementary Fig. S9). Nearing the end of the time course additional lower molecular weight bands are observed which we interpret to be degradation of the S1 subunit. Regardless of whether wild-type or prefusion-stabilized S was used or whether ACE2 was bound to the S ectodomains, there is no prominent band that corresponds to a S2′ cleavage product (approximately 52 kDa).

**Figure 5:**
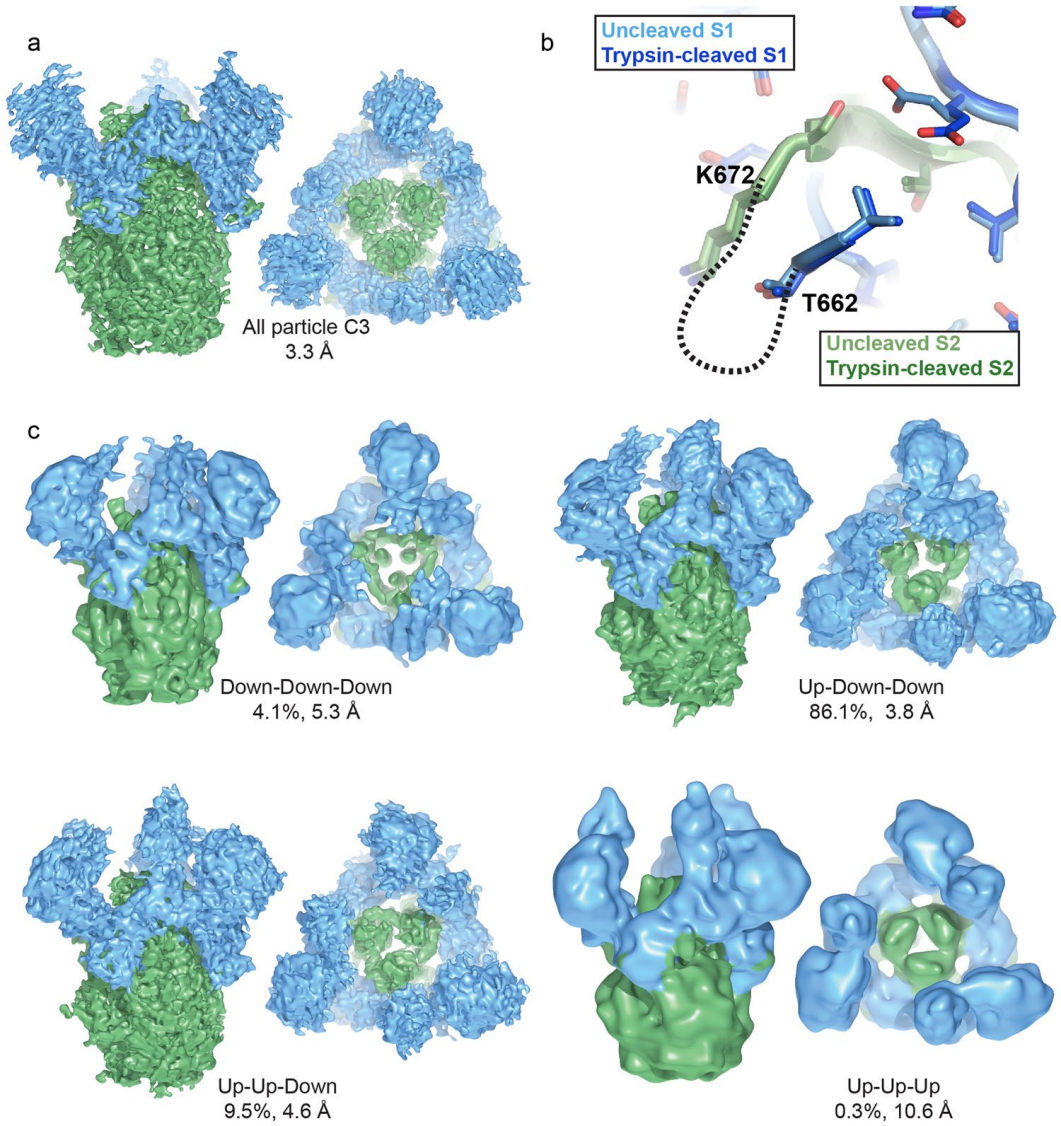
Trypsin cleavage does not impart conformational changes in prefusion SARS-CoV S 2P spike. (**a**) An all particle reconstruction of trypsin-cleaved SARS-CoV S 2P with C3 symmetry. (**b**) Comparison of the trypsin-cleaved and uncleaved SARS-CoV S 2P coordinate models reveals no differences in protein structure near the cleavage site. The last residue in S1 (T662) and the first residue in S2 (K672) visualized in the coordinates are labeled. (**c**) Classification of the heterogeneous S1 RBD in the trypsin-cleaved S shows a predominance of the single-‘up’ conformation as well as the observation of an all-‘down’ conformation not seen in the uncleaved S 2P sample. Colors are as in Fig. 1.

To analyze the cleavage products in detail, we performed cryo-EM analysis on the trypsin-cleaved SARS-CoV S 2P ectodomain. Using all-particles and C3 symmetry yielded a reconstruction at 3.3 Å resolution (Fig. 5, Supplementary Tables S1, S4 and Fig. S10). The short loop containing the S1/S2 cleavage site is disordered in the uncleaved S 2P reconstruction and remains disordered in the S 2P trypsin cleaved reconstruction. Moreover, examination of the structure models indicates no significant differences between the trypsin-cleaved and uncleaved SARS-CoV S 2P (Fig. 5b). Fine sorting of S1 RBD positions of the trypsin-cleaved S reveals a very similar distribution of ‘up’ S1 RBD conformations available for receptor binding as in the uncleaved samples, although we additionally observe a small proportion of S1 RBD in the all-‘down’ conformation (Fig. 5c). These results indicate that trypsin-cleavage at S1/S2 does not impart large conformational changes on the prefusion-stabilized SARS-CoV S 2P and justifies the removal of S1/S2 cleavage sites for the production of more homogeneous material as vaccine immunogens.

**Figure 6:**
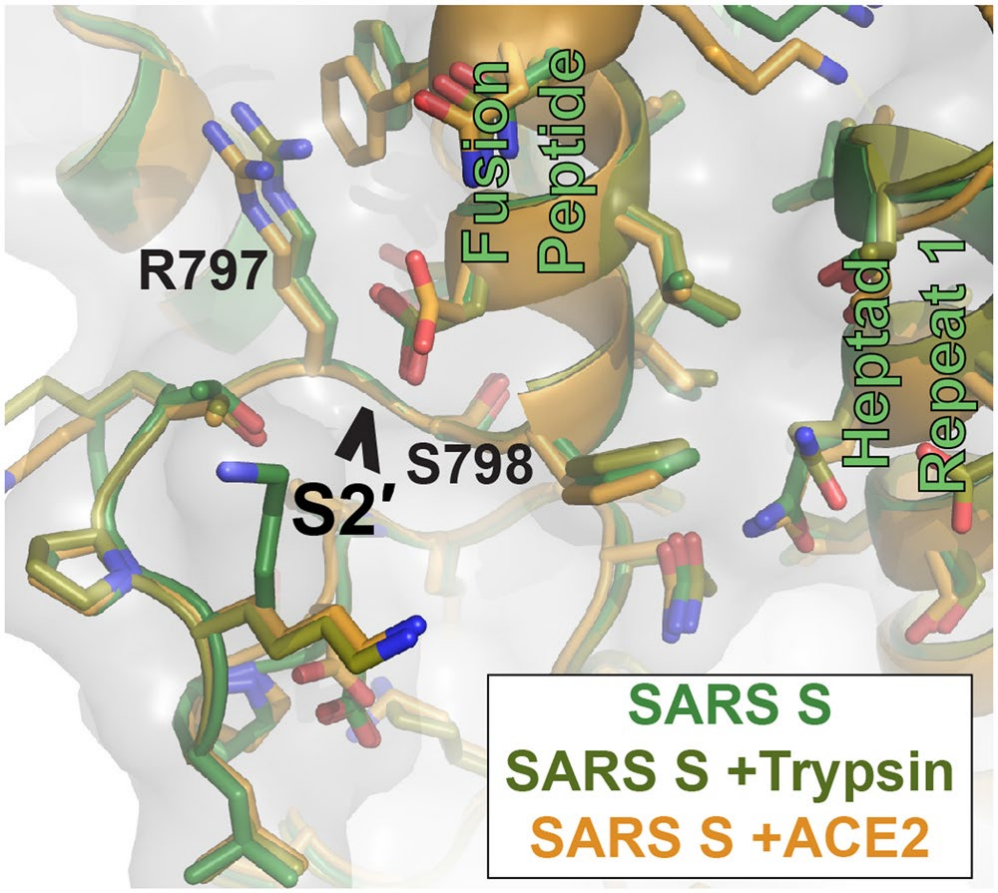
S2′ secondary cleavage site is occluded. Superimposition of the SARS-CoV S 2P, uncleaved, cleaved and ACE2-bound structures reveals no structural changes within this region. The S2**′** site after amino acid R797 is shielded from cleavage by an adjacent loop and the fusion peptide which packs against an N-terminal region of S2 HR1.

In agreement with the *in vitro* limited proteolysis experiments, the S2′ site remains uncleaved in the trypsin-cleaved S 2P ectodomain structures. The arginine at the proposed S2′ cleavage site in all coronavirus spike structures published to date and presented here is blocked from cleavage by a loop protruding from just N-terminal to the fusion peptide and by an N-terminal helix of S2 HR1 (Fig. 6). Exposure of this site for cleavage may require remodeling of this penultimate loop or HR1 beyond the conformation observed in the prefusion state and hence possibly not accessible in our prefusion-stabilized S 2P ectodomains.

## Discussion

Here, we present the first structures of a trimeric coronavirus spike ectodomain bound to a protein receptor as well as the first structures of prefusion S1/S2 cleaved coronavirus spikes. Our structures of the SARS-CoV S 2P ectodomain bound to a soluble form of human ACE2 receptor show that any conformational changes induced in S by receptor binding are more likely to be due to the disruption of protein-protein interactions rather than the formation of additional contacts between the S1 RBD and other regions of S (Supplementary Fig. S8). The only conformational change that we observe in the ACE2-SARS-CoV S 2P structures is the transition to a short 3_10_-helix at the top of the S2 central α-helix when uncapped by receptor-bound S1 RBD suggesting that more extensive conformational changes may be initiated here. 3_10_-helices have been hypothesized to act as intermediates in coil-to-α-helix transitions^32^. Transitions between 3_10_-helices and α-helices has also been hypothesized for the voltage sensing domains of potassium channels where it has been proposed that the transition plays a role in changing from a resting or activated state to a relaxed state^33^. Though more extensive changes are likely to drive conformational rearrangements accompanying membrane fusion, the 2P prefusion stabilizing mutations adjacent to the short 3_10_-helix may block these changes from occurring. However, both wild-type and 2P SARS-CoV S ectodomains bound to ACE2 were cleaved equally by trypsin, indicating that at least at the level of trypsin protease susceptibility, these two receptor-bound complexes appear equivalent.

Strategies similar to those we have used have been employed to stabilize HIV envelope glycoprotein (Env) trimers in prefusion conformation^34,35^. HIV Env, which, like SARS CoV S also engages a protein receptor, CD4, undergoes a large conformational change on the viral surface upon binding^36^. Similarly, the soluble, stabilized versions of Env undergo large conformational changes upon CD4 binding^37–39^. Conversely, the Ebola virus glycoprotein (GP) undergoes only modest conformational changes upon binding its receptor, NPC-1^40,41^. Thus, these class I fusion machines likely have fundamental differences in the fusion process that requires further study.

These structural and biochemical data show that the introduction of the 2P prefusion-stabilizing mutations does not interfere with receptor binding or recognition by trypsin-like proteases. Our structural examinations of both receptor-bound and trypsin-cleaved prefusion-stabilized SARS-CoV S 2P proteins indicate that neither receptor binding nor trypsin cleavage induce large conformational changes in stabilized spikes. Additionally, these structures and accompanying limited proteolysis experiments show that neither receptor binding nor trypsin cleavage expose the S2′ cleavage site for proteolysis within the stabilized spike ectodomains. This work increases the body of knowledge surrounding these prefusion-stabilized spikes as presenting native surfaces for interactions with host factors while maintaining their prefusion conformation.

## Methods

### Plasmid construction, Protein expression and purification

A mammalian-codon-optimized gene encoding SARS-CoV S (Tor2 strain) residues 1–1190 with a C-terminal T4 fibritin trimerization domain, an HRV3C cleavage site, an 8xHis-tag and a Twin-Strep-tag was synthesized and subcloned into the eukaryotic-expression vector pαH. Proline-substituted variant harboring K968P and V969P mutations was generated based on this construct. The resulting plasmids, designated SARS S WT and SARS S 2P respectively, were transfected into 1 L FreeStyle 293-F cells (Life Technologies). 293-F cells were used without validation and were not tested for mycoplasma. Three hours after transfection, kifunensine was added to a final concentration of 5 μM. Cultures were harvested after 6 d, and protein was purified from the supernatant using Strep-Tactin resin (IBA). HRV3C protease (1% wt/wt) was added to the protein and the reaction was incubated overnight at 4 °C. The digested protein was further purified using a Superose 6 16/70 column (GE Healthcare Life Sciences).

A gene encoding human ACE2 residues 1-615 with an HRV3C cleavage site, an 8xHis-tag and a Twin-Strep-tag was synthesized and subcloned into the eukaryotic-expression vector pαH. This protein was expressed as described above for S proteins. The HRV3C digested ACE2 protein was further purified using a Superdex 200 column (GE Healthcare Life Sciences).

To make complexes of SARS S 2P with soluble ACE2, the two proteins were combined in a 1:1.1 molar ratio prior to overnight incubation and size-exclusion chromatography on a Superose 6 Increase 10/300 (GE Healthcare Life Sciences).

### Surface plasmon resonance

Purified SARS-CoV S WT or SARS-CoV S 2P ectodomain was captured on an NTA sensor chip via an 8X HisTag to a level of ∼330 response units (RU) per cycle using a Biacore X100 (GE Healthcare). The NTA sensorchip was regenerated between cycles using 350 mM EDTA and 100 mM NaOH followed by 0.5 mM NiCl_2_. A sample containing 10 mM HEPES pH 8.0, 150 mM sodium chloride and 0.05% Surfactant P20 (GE Healthcare) (HBS-P+) was injected over the SARS-CoV S and reference flow cells, followed by injections of purified ACE2 serially diluted 2-fold from 100 nM to 6.25 nM in HBS-P+, with a duplication of the 25 nM concentration. The data were double-reference subtracted and fit to a 1:1 binding model using BIAevaluation analysis software.

### Electron microscopy data collection

3 µL of 0.47 mg/mL of protein or protein complexes were mixed with 1 µL of 0.04% (w/v) amphipol A8-35 (Anatrace) just prior to grid preparation. 3 µL of the protein mixture, final concentration 0.35 mg/mL, was spotted on to plasma cleaned C-flat grids with a 2 µm/2 µm spacing. Grids were blotted for 3 seconds before plunge freezing in liquid ethane on a Vitrobot. Grids were loaded onto a Titan Krios and data was collected using Leginon^42^ at a total dose of 65 e^−^/Å^2^. Frames were aligned with MotionCor2 (UCSF)^43^ implemented in the Appion workflow^44^. Particles were selected using DoG picker^45^. Images were assessed and particle picks were masked using EM Hole Punch^46^. The CTF for each image was estimated using Gctf ^47^.

### Electron microscopy data processing

Initial particle stacks were cleaned using multiple rounds of 2D classification in RELION^48^. Good particles were selected as resembling prefusion coronavirus spikes. For the SARS S 2P and trypsin-treated SARS S 2P, all particles from the clean stacks were used for reconstruction with C3 symmetry. All datasets were extensively sorted using 3D classification to examine heterogeneity in the S1 RBDs as described previously^17^. Briefly, 3D masks were defined to encompass the possible heterogeneity at each S1 RBD position. The density within these masks was then removed from unfiltered, unsharpened reconstructions. We then used relion_project with image subtraction to create a particle stack containing only the signal arising from the masked density. Finally, we used focused 3D classification to identify compositional and conformational states at each S1 RBD position. All 3D reconstructions were produced with RELION^48^ and final refinements were performed with a six-pixel soft-edge solvent mask. Post-processing was applied to each reconstruction to apply B-factor sharpening and amplitude corrections as well as to calculate local resolution maps.

Coordinate models were built for several of the high-resolution reconstructions using 5I08.pdb^16^, 2AJF.pdb^23^ and 5X4S pdb^22^ as template models with reference to a recently published wild-type SARS S ectodomain (5X58 pdb^22^). Manual model building was performed in Coot^49^ with coordinate refinement in Rosetta Relax^50^ and real-space refinement in PHENIX^51^ with final rounds of coordinate refinement and ADP estimation performed in PHENIX.

### Limited proteolysis

10 µg of either SARS-CoV S 2P or SARS-CoV S WT ectodomains were mixed with soluble ACE2 in a 1:1.1 ratio and incubated overnight at 4 °C. Proteins were digested with 0.1% (w/w) TPCK trypsin at room temperature. For SDS-PAGE analysis, samples were removed at indicated time points, immediately mixed with SDS loading buffer and incubated at 95 °C for 1 minute. For EM analysis, the proteolysis reaction was stopped after two hours using phenyl-methyl-sulfonyl-floride (PMSF) at a final concentration of 1 mM and then immediately loaded onto a Superose 6 Increase 10/300 size-exclusion column.

## Electronic supplementary material

Supplementary Information

## Data Availability

Electron microscopy maps are deposited in the Electron Microscopy Data Bank (www.ebi.ac.uk/pdbe/emdb/) with accession codes: 7573, 7574, 7575, 7576, 7577, 7578, 7579, 7580, 7581, 7582, 7584, 7585, 7586, 7601, 7602, 7603, 7604, 7605, 7606, 7607 and 7608. Atomic models for select maps are deposited in the Protein Data Bank (www.rcsb.org/) with accession codes 6CRV, 6CRW, 6CRX, 6CRZ, 6CS0, 6CS1 and 6CS2.
