# Publisher Correction: Stabilized coronavirus spikes are resistant to conformational changes induced by receptor recognition or proteolysis

**DOI:** 10.1038/s41598-018-36918-8

**Published:** 2018-12-10

**Authors:** Robert N. Kirchdoerfer, Nianshuang Wang, Jesper Pallesen, Daniel Wrapp, Hannah L. Turner, Christopher A. Cottrell, Kizzmekia S. Corbett, Barney S. Graham, Jason S. McLellan, Andrew B. Ward

**Affiliations:** 1 Department of Integrative Structural and Computational Biology, The Scripps Research Institute, La Jolla, CA 92037 USA; 2 Department of Biochemistry and Cellular Biology, Geisel School of Medicine at Dartmouth, Hanover, NH 03755 USA; 3 Present Address: Department of Molecular Biosciences, The University of Texas at Austin, Austin, TX 78712 USA; 4 Vaccine Research Center, National Institute of Allergy and Infectious Diseases, Bethesda, MD 20814 USA

Correction to: *Scientific Reports* 10.1038/s41598-018-34171-7, published online 24 October 2018

The original version of this Article contained an error in Affiliation 2, which was incorrectly given as ‘Department of Biochemistry and Cellular Biology, Geisel School of Medicine at Dartmouth, Hanover, NH, 03755, Germany’. The correct affiliation is listed below:

Department of Biochemistry and Cellular Biology, Geisel School of Medicine at Dartmouth, Hanover, NH, 03755, USA’

This error has now been corrected in the HTML and PDF versions of the Article.

