# Supplementary material for: Stabilized coronavirus spikes are resistant to conformational changes induced by receptor recognition or proteolysis
Source: Sci Rep. 2018 Oct 24;8:15701. doi: 10.1038/s41598-018-34171-7 (PMC6200764; doi:10.1038/s41598-018-34171-7)
Supplement: Supplementary file 1 — Supplementary Information [file 41598_2018_34171_MOESM1_ESM.pdf]

Supplementary Information

Stabilized coronavirus spikes are resistant to conformational changes induced by receptor recognition or proteolysis

Robert N. Kirchdoerfer[1], Nianshuang Wang[2,3], Jesper Pallesen[1], Daniel Wrapp[2,3], Hannah L. Turner[1], Christopher A. Cottrell[1], Kizzmekia S. Corbett[4], Barney S. Graham[4], Jason S. McLellan[2,3], Andrew B. Ward[1#]

[1]Department of Integrative Structural and Computational Biology, The Scripps Research Institute, La Jolla, CA 92037

[2]Department of Biochemistry and Cellular Biology, Geisel School of Medicine at Dartmouth, Hanover, NH 03755

[3]Current address: Department of Molecular Biosciences, The University of Texas at Austin, Austin, TX, 78712

[4]Vaccine Research Center, National Institute of Allergy and Infectious Diseases, Bethesda, MD 20814

[#]Corresponding author

ABW: andrew@scripps.edu

	SARS S 2P, one 'up'	SARS S 2P, two 'up'	SARS S 2P, three 'up'	SARS S 2P, all particle	SARS S 2P Trypsin, all 'down'	SARS S 2P Trypsin, one 'up'	SARS S 2P Trypsin, two 'up'	SARS S 2P Trypsin, three 'up'	SARS S 2P Trypsin, all particle
EMDB	7574	7575	7576	7573	7581	7578	7579	7580	7577
PDB	6CRW	6CRX	-	6CRV	-	6CS0	6CS1	-	6CRZ
Defocus Range (μm)	-1.2 – -2.2	-1.2 – -2.2	-1.2 – -2.2	-1.2 – -2.2	-1.2 – -2.2	-1.2 – -2.2	-1.2 – -2.2	-1.2 – -2.2	-1.2 – -2.2
Symmetry	C1	C1	C3	C3	C3	C1	C1	C3	C3
Frames/movie	48	48	48	48	48	48	48	48	48
Initial particles	104,184	104,184	104,184	104,184	162,177	162,177	162,177	162,177	162,177
Final particles	58,349	38,603	3,316	104,184	6,664	139,746	15,314	452	162,177
Map resolution (Å)	3.9	3.9	4.5	3.2	5.3	3.8	4.6	10.6	3.3
B-factor sharp ($Å^2$)	-94	-109	-93	-123	-145	-127	-139	-458	-139

Table 1. Electron microscopy data and refinement statistics. All image movies were collected at a nominal magnification of 29,000, resulting in an image pixel size of 1.03 Å on a Titan Krios operating at 300 keV with a total 65 $e^-/Å^2$ using 250 ms/frame. Final resolution was determined at a Fourier shell correlation of 0.143 between independently refined half maps.

	SARS S 2P + ACE2, S1 RBD configuration: ‘ up’ , ‘ up’ , ‘ down’	SARS S 2P + ACE2, S1 RBD configuration: ‘ up’ , ‘ down’ , ‘ down’	SARS S 2P + ACE2, S1 RBD configuration: ‘ ACE2, ‘ down’ , ‘ down’	SARS S 2P + ACE2, S1 RBD configuration: ‘ ACE2, ‘ down’ , ‘ up’	SARS S 2P + ACE2, S1 RBD configuration: ‘ ACE2, ‘ up’ , ‘ down’	SARS S 2P + ACE2, S1 RBD configuration: ‘ ACE2, ‘ up’ , ‘ up’	SARS S 2P + ACE2, S1 RBD configuration: ‘ ACE2, ‘ ACE2’ , ‘ down’	SARS S 2P + ACE2, S1 RBD configuration: ‘ ACE2, ‘ ACE2’ , ‘ up’	SARS S 2P + ACE2, S1 RBD configuration: ‘ ACE2, ‘ ACE2’ , ‘ ACE2’
EMDB	7584	7585	7586	7601	7602	7603	7604	7605	7606
PDB	-	-	-	-	-	-	-	-	-
Defocus Range (µm)	-1.2 - -2	-1.2 - -2	-1.2 - -2	-1.2 - -2	-1.2 - -2	-1.2 - -2	-1.2 - -2	-1.2 - -2	-1.2 - -2
Symmetry	C1	C1	C1	C1	C1	C1	C1	C1	C3
Frames/movie	24	24	24	24	24	24	24	24	24
Initial particles	164,426	164,426	164,426	164,426	164,426	164,426	164,426	164,426	164,426
Final particles	19,102	63,343	22,916	6,755	23,854	938	7,584	3,759	965
Map resolution (Å)	6.6	4.4	6.2	7.6	5.5	13.2	8.1	8.8	9.3
B-factor sharp (Å^2)	-144	-161	-155	-215	-127	-725	-458	-466	-590

Table 2. Electron microscopy data and refinement statistics, continued. All image movies were collected at a nominal magnification of 29,000, resulting in an image pixel size of 1.03 Å on a Titan Krios operating at 300 keV with a total 65 e^-/Å^2 using 250 ms/frame. Final resolution was determined at a Fourier shell correlation of 0.143 between independently refined half maps.

	SARS S 2P + ACE2, all ACE2 bound particles	SARS S 2P + ACE2, S1 RBD configuration: ‘ ACE2’ , ‘ up/down’ , ‘ ’ down’	SARS S 2P + ACE2, RBD-ACE2 focused refinement
EMDB	7582	7607	7608
PDB	6CS2	-	-
Defocus Range (μm)	-1.2 - -2	-1.2 - -2	-1.2 - -2
Symmetry	C1	C1	C1
Frames/movie	24	24	24
Initial particles	164,426	164,426	164,426
Final particles	66,771	46,770	52,916
Map resolution (Å)	4.4	4.6	7.9
B-factor sharp (Å^2)	-125	-134	-726

Table 3. Electron microscopy data and refinement statistics, continued. All image movies were collected at a nominal magnification of 29,000, resulting in an image pixel size of 1.03 Å on a Titan Krios operating at 300 keV with a total 65 e^-/Å^2 using 250 ms/frame. Final resolution was determined at a Fourier shell correlation of 0.143 between independently refined half maps.

	SARS S 2P, all particles	SARS S 2P, one S1 RBD ‘ up’	SARS S 2P, two S1 RBD ‘ up’	SARS S 2P Trypsin, all particles	SARS S 2P Trypsin, one S1 RBD ‘ up’	SARS S 2P Trypsin, two S1 RBD ‘ up’	SARS S 2P + ACE2, all ACE2 bound particles
**Refinement**							
PDB	6CRV	6CRW	6CRX	6CRZ	6CS0	6CS1	6CS2
EMDB	7573	7574	7575	7577	7578	7579	7582
Initial model used (PDB code)	5I08, 5X4S	6CRV, 2AJF	6CRW	6CRV	6CRW	6CRX	6CRV, 2AJF
Model composition							
Non-hydrogen atoms	21,036	25,994	26,012	21,078	24,909	25,995	27,860
Protein residues	20,499	25,015	25,015	20,499	25,024	25,008	26,679
Ligands	537	979	997	579	885	987	1,181
B factors (Å^2)	62	144	143	60	118	188	269
Protein	61	141	140	59	116	186	271
Ligand	126	221	206	90	164	242	237
EMRinger	3.13	1.27	1.86	3.40	1.18	0.43	0.88
R.m.s. deviations							
Bond lengths (Å)	0.013	0.008	0.010	0.008	0.007	0.006	0.007
Bond angles (°)	1.34	1.23	1.34	1.20	1.26	1.17	1.20
Validation							
MolProbity score	1.50	1.76	1.83	1.43	1.85	1.86	1.87
Clashscore	4.4	6.7	7.0	3.1	6.8	7.3	7.6
Non-Rotamers (%)	0.3	0.1	0.2	0.1	0.0	0.0	0.2
Ramachandran plot							
Favored (%)	95.86	94.28	93.17	95.28	92.22	92.82	92.93
Allowed (%)	4.14	5.69	6.80	4.72	7.78	7.18	7.01
Outliers (%)	0.00	0.03	0.03	0.00	0.00	0.00	0.06

Table 4. Coordinate refinement. Coordinate models were refined in Phenix with atomic-displacement parameter refinement in the final round[1]. R.m.s. deviations, Molprobity scores, clash scores, rotamer assessment and Ramachandran statistics were calculated with Molprobity[2]. EMRinger scores were calculated using EMRinger[3].

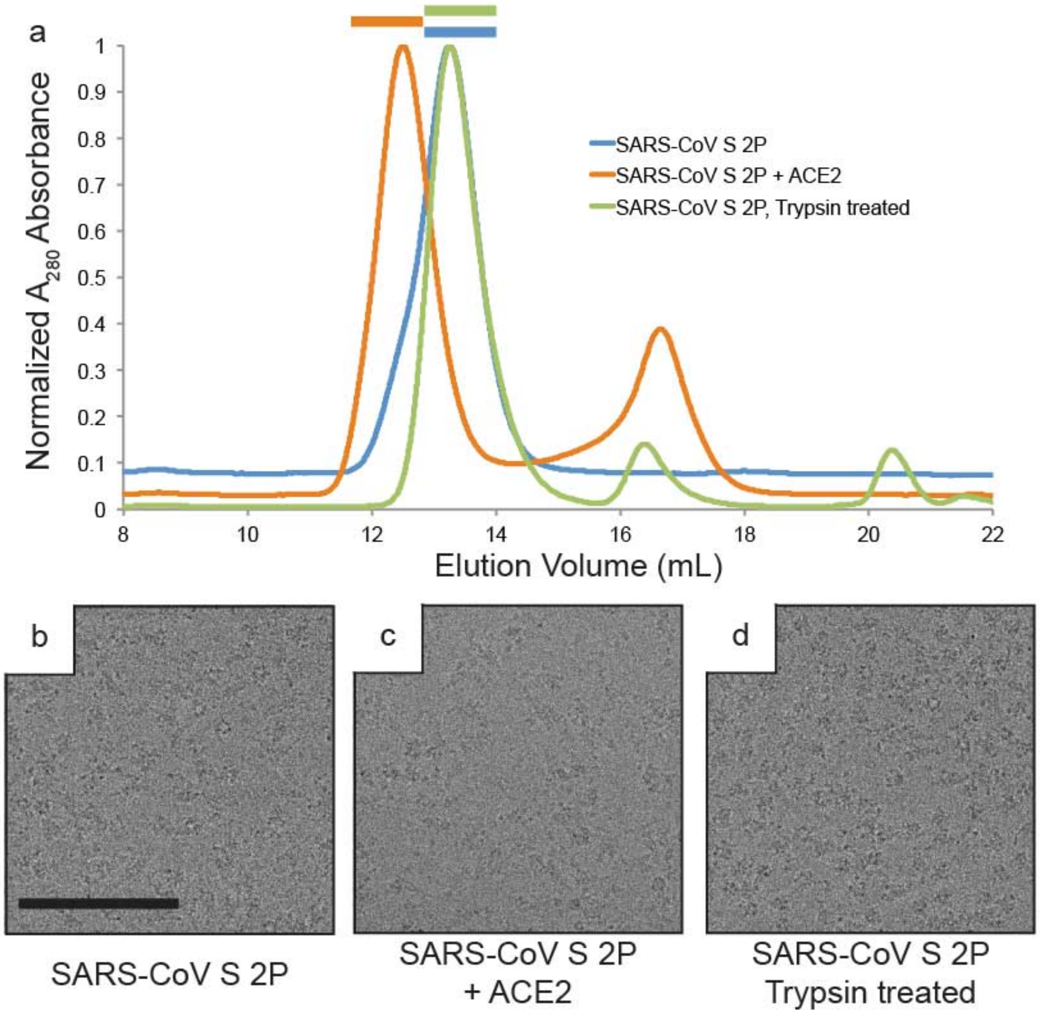

Supplemental Figure 1: Purification of SARS-CoV S 2P proteins used for study. a) Size exclusion traces of purified proteins and protein complexes on a Superose6 increase 10/300 column. Fractions taken for electron microscopy sample preparation are indicated by the colored bars above the graph. b-d) Representative electron micrographs for each protein sample used in this study. Scale corresponds to 100 nm.

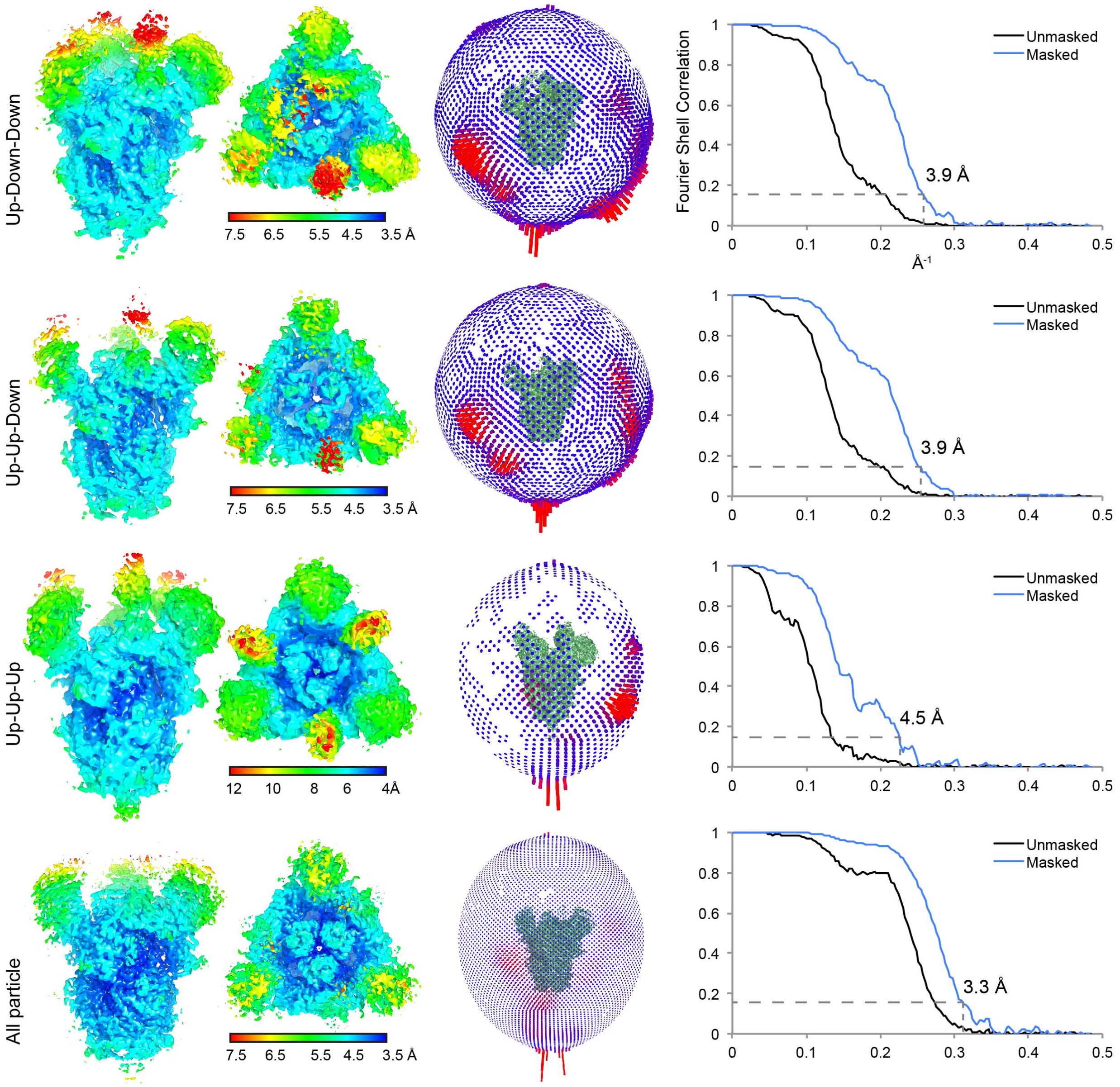

Supplemental Figure 2: Local Resolution, angular distribution and Fourier shell correlation plots for structures derived from the SARS S 2P dataset. Local resolution of each reconstruction (side and membrane distal views) was calculated in RELION[4] (left). Angular distribution was taken from the relion_refine output (center). Fourier shell correlation (FSC) curves were calculated by relion_postprocess with and without a low-pass filtered mask that had been extended by three pixels with a six pixel Gaussian soft edge (right).

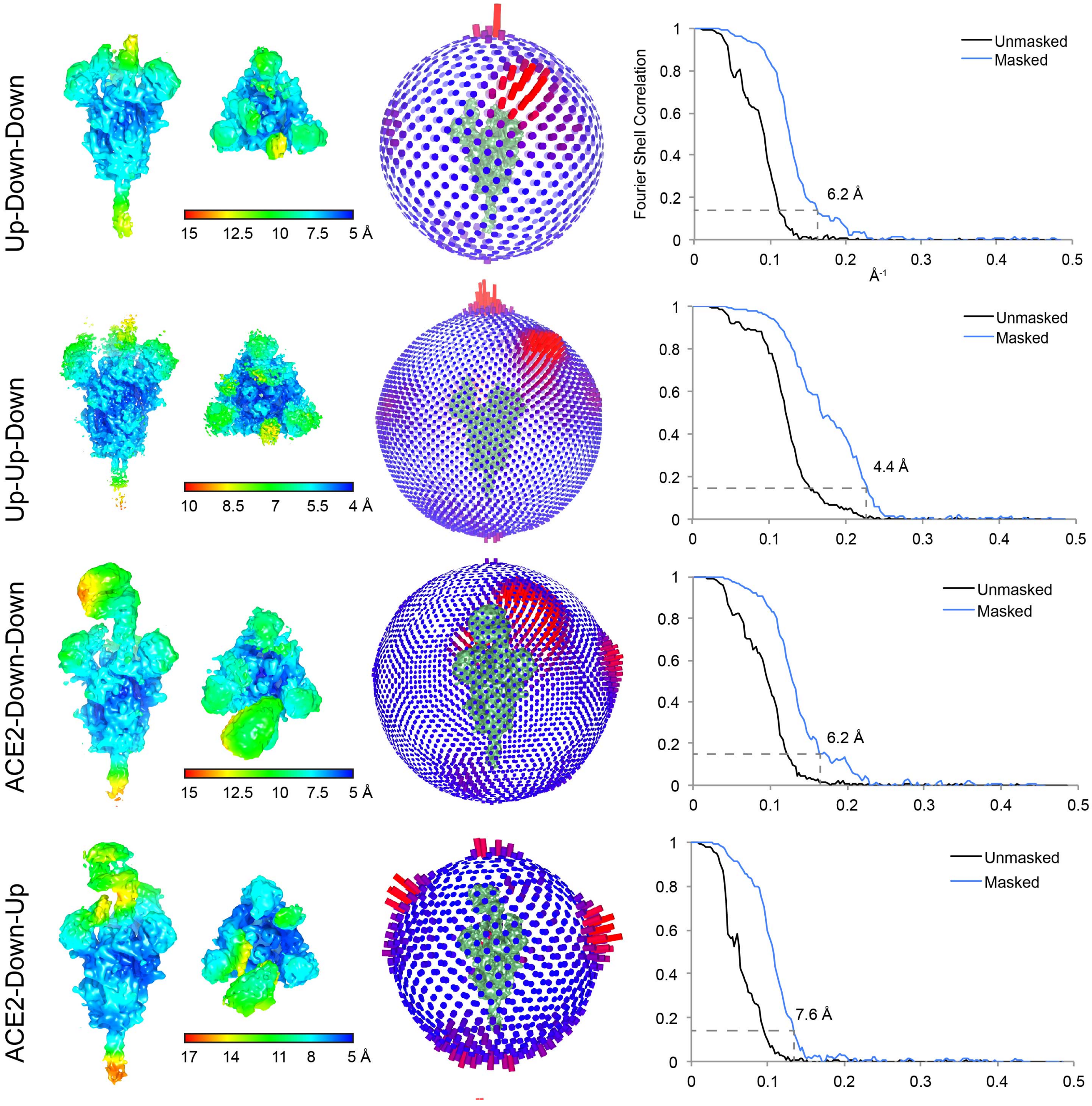

Supplemental Figure 3: Local Resolution, angular distribution and Fourier shell correlation plots for structures derived from the SARS S 2P – ACE2 dataset (1/3). Local resolution of each reconstruction (side and membrane distal views) was calculated in RELION[4] (left). Angular distribution was taken from the relion_refine output (center). Fourier shell correlation (FSC) curves were calculated by relion_postprocess with and without a low-pass filtered map that had been extended by three pixels with a six pixel Gaussian soft edge (right).

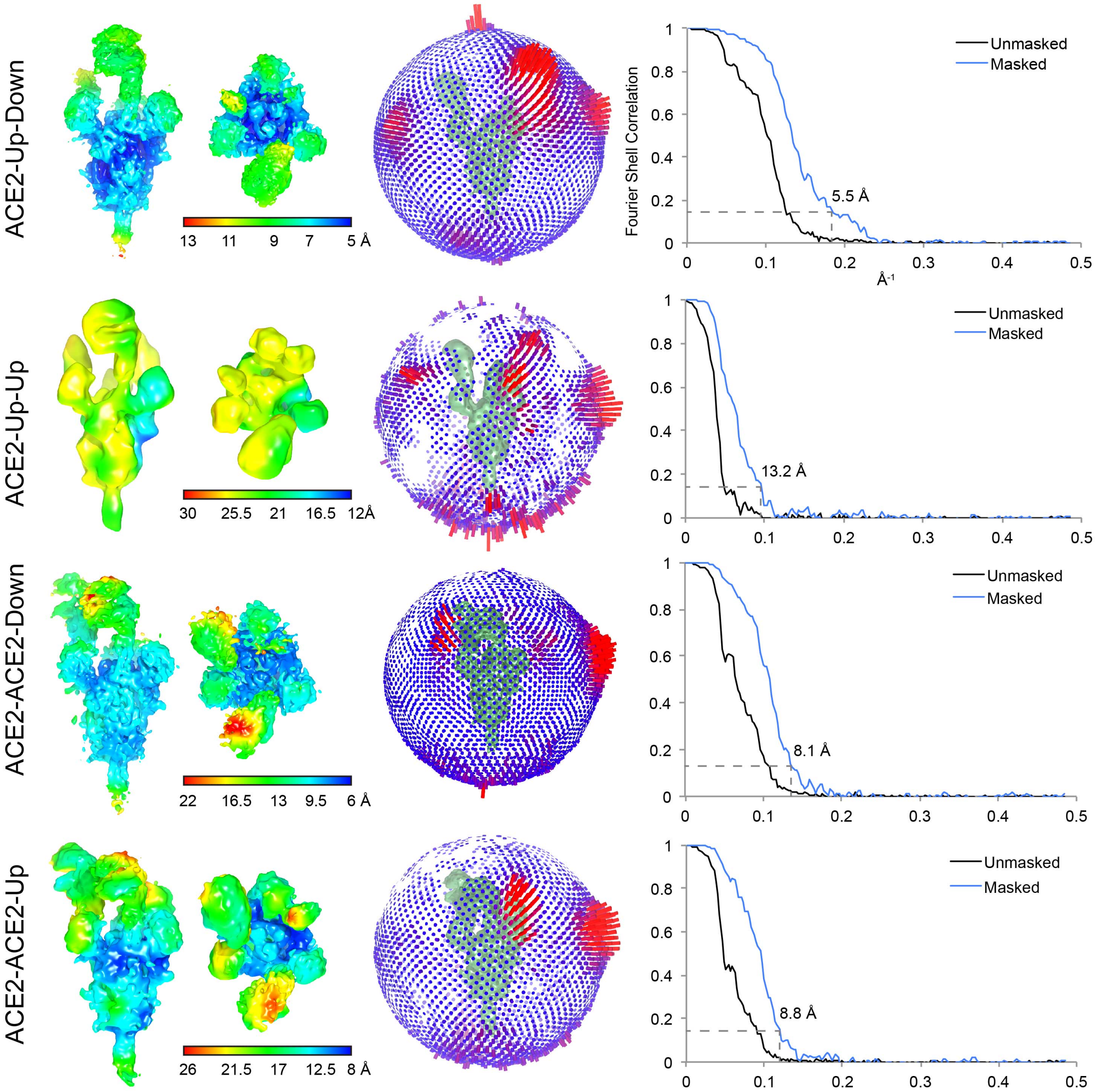

Supplemental Figure 4: Local Resolution, angular distribution and Fourier shell correlation plots for structures derived from the SARS S 2P – ACE2 dataset (2/3). Local resolution of each reconstruction (side and membrane distal views) was calculated in RELION[4] (left). Angular distribution was taken from the relion_refine output (center). Fourier shell correlation (FSC) curves were calculated by relion_postprocess with and without a low-pass filtered map that had been extended by three pixels with a six pixel Gaussian soft edge (right).

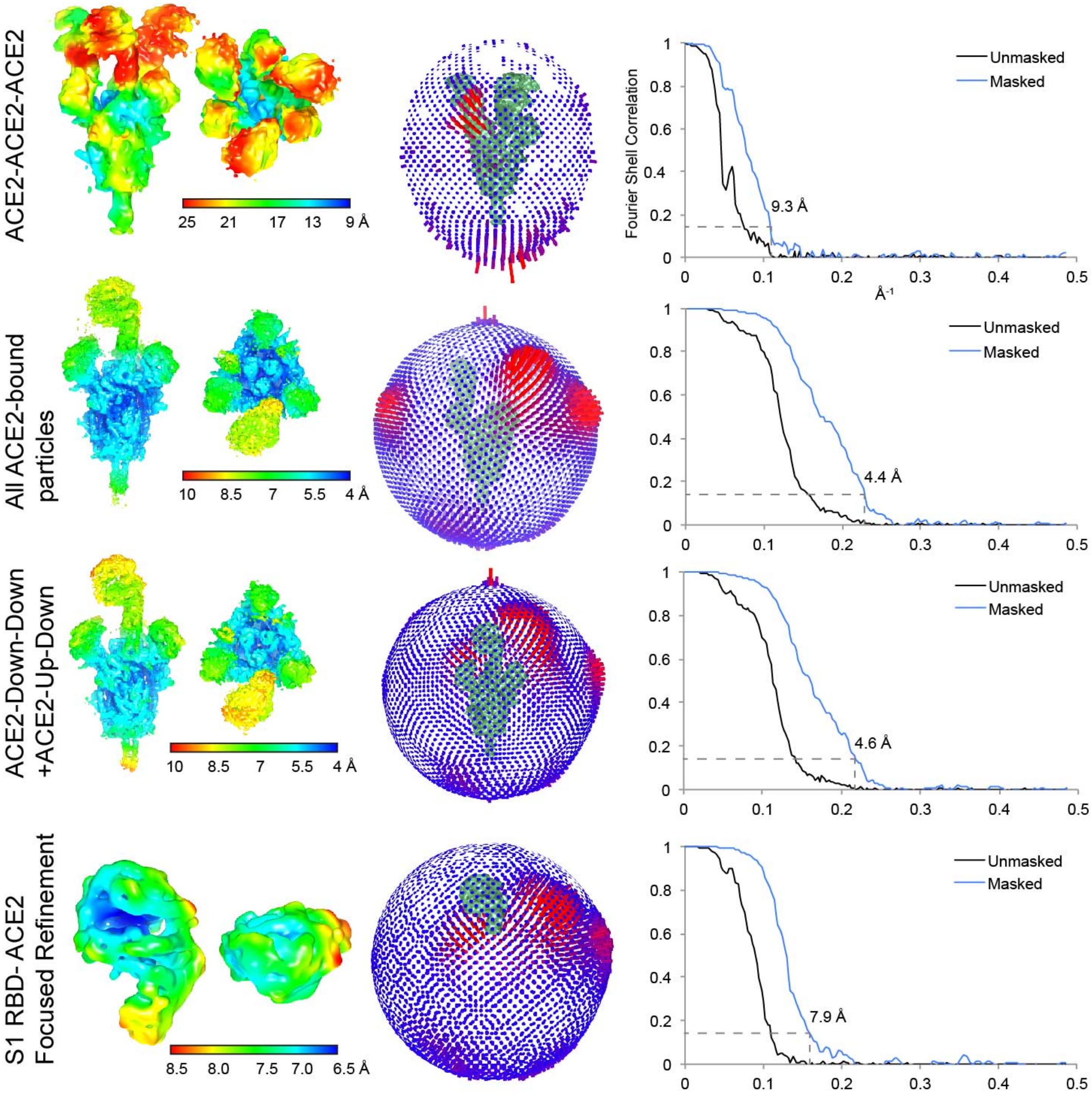

Supplemental Figure 5: Local Resolution, angular distribution and Fourier shell correlation plots for structures derived from the SARS S 2P – ACE2 dataset (3/3). Local resolution of each reconstruction (side and membrane distal views) was calculated in RELION[4] (left). Angular distribution was taken from the relion_refine output (center). Fourier shell correlation (FSC) curves were calculated by relion_postprocess with and without a low-pass filtered map that had been extended by three pixels with a six pixel Gaussian soft edge (right).

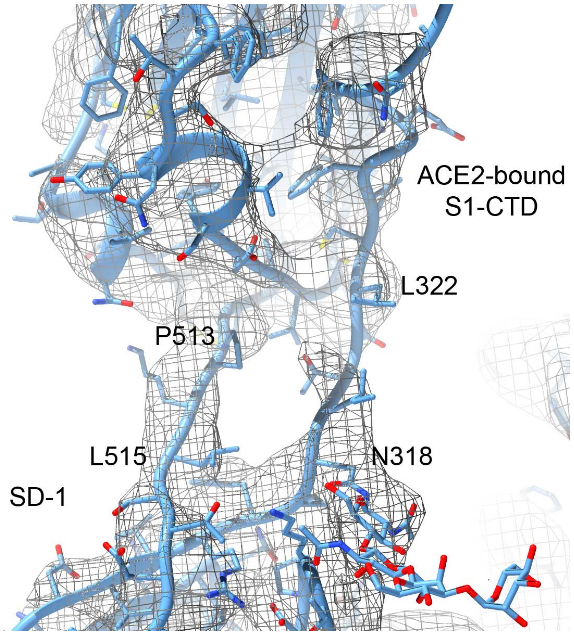

Supplemental figure S6: EM Density in a hinge region of SARS-CoV S 2P. There is ordered density between the S1 CTD and the sub-domain 1 (SD-1) in the ACE2-bound S protomer (6CS2.pdb and EMD-7582) to support the positioning of the S1 CTD -ACE2 complex (2AJF.pdb[5]).

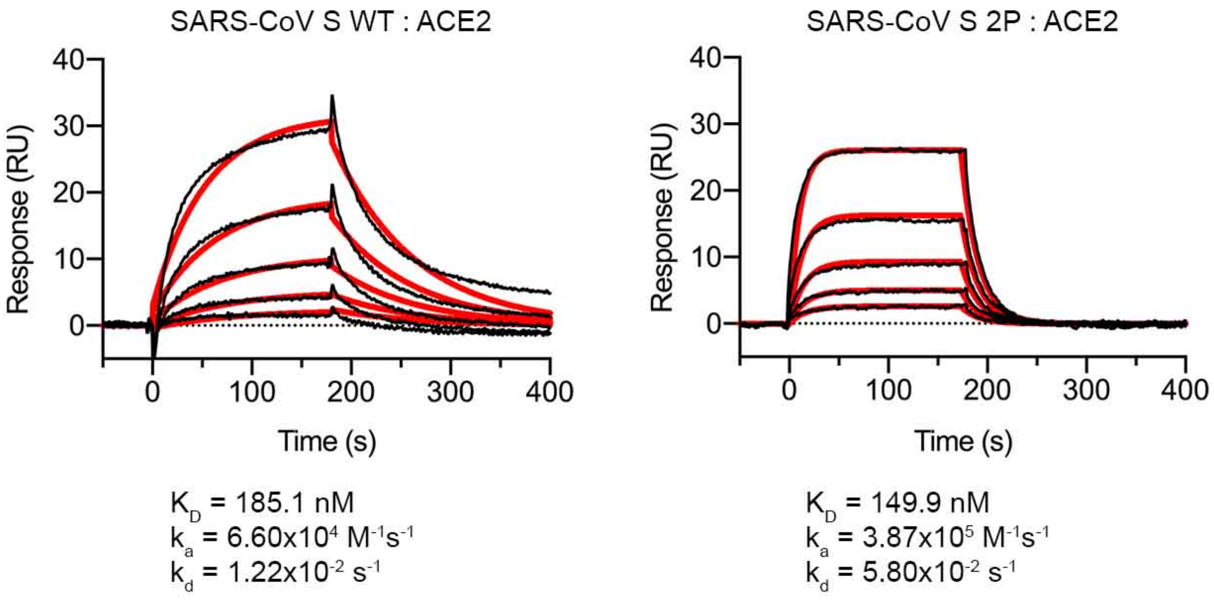

Supplementary Figure S7: ACE2-binding affinities for SARS-CoV S ectodomains. SARS-CoV spike ectodomains were immobilized on a NTA sensor chip and dilutions of soluble ACE2 were injected to examine the binding affinity and kinetics of each spike ectodomain with ACE2.

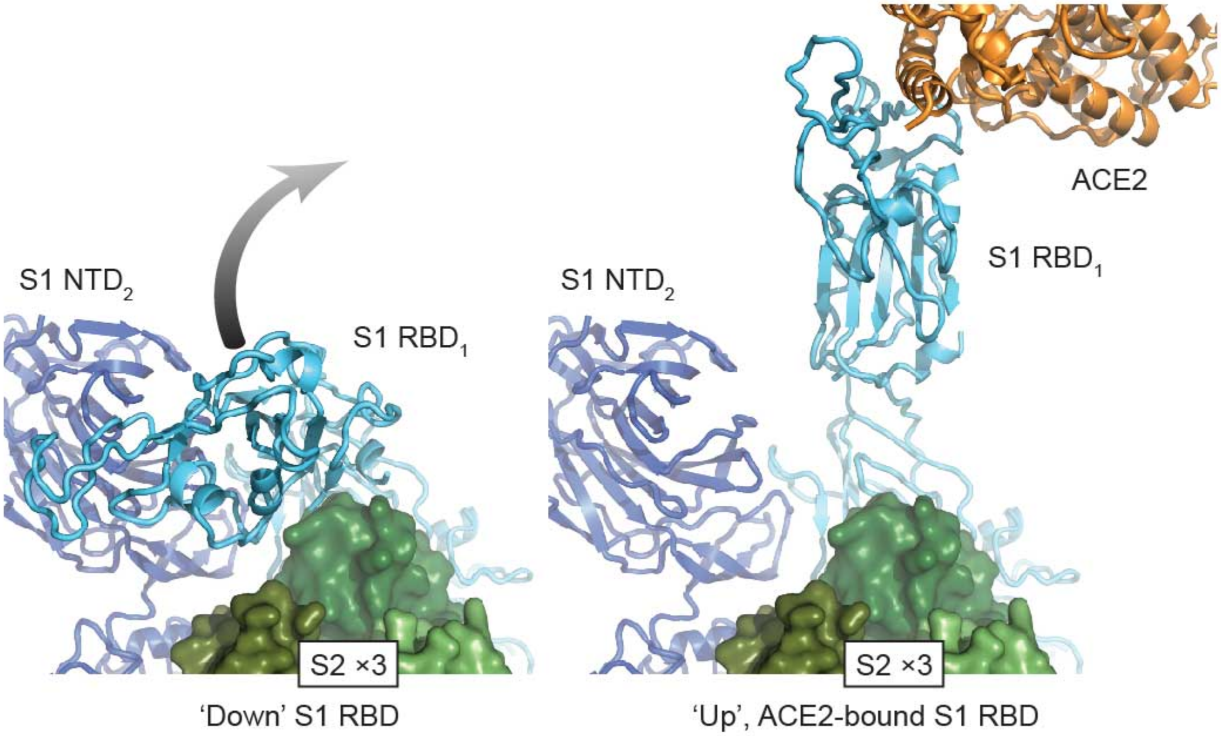

Supplementary Figure 8: Comparison of 'up' and 'down' S1 RBD conformations. The S1 RBD in the downward conformation is wedged between an adjacent protomer's S1 NTD and S2 (left). Upon transitioning to the upward conformation (right), the S1 RBD looses its interactions with the adjacent protomer and gains the ability to bind to host protein receptor, ACE2. Adjacent S1 protomers have been omitted for clarity.

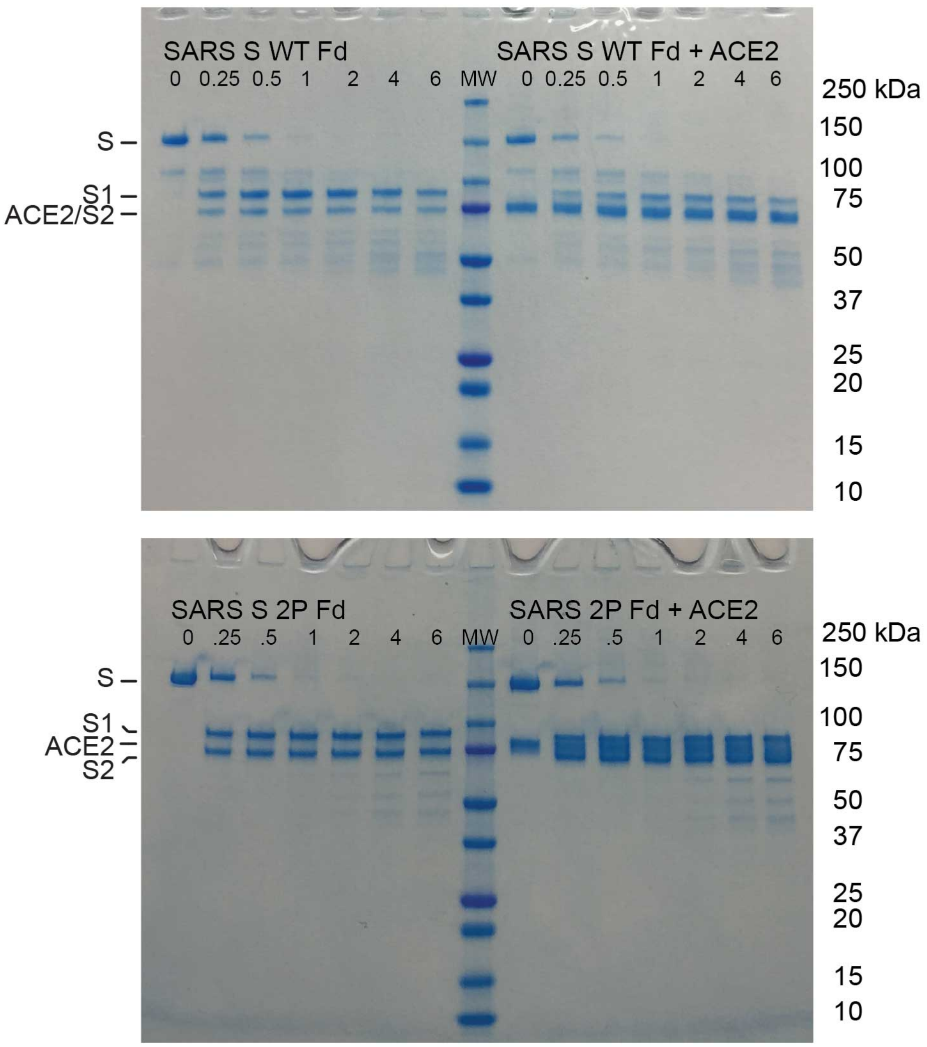

Supplemenatry Figure 9: Limited proteolysis of SARS coronavirus spike ectodomains with trypsin. SARS S WT or 2P was incubated with or without soluble ACE2 and then digested with 0.1% trypsin at 20˚C for the time indicated above each lane (hours). Molecular weight labels for the protein standards are included at right.

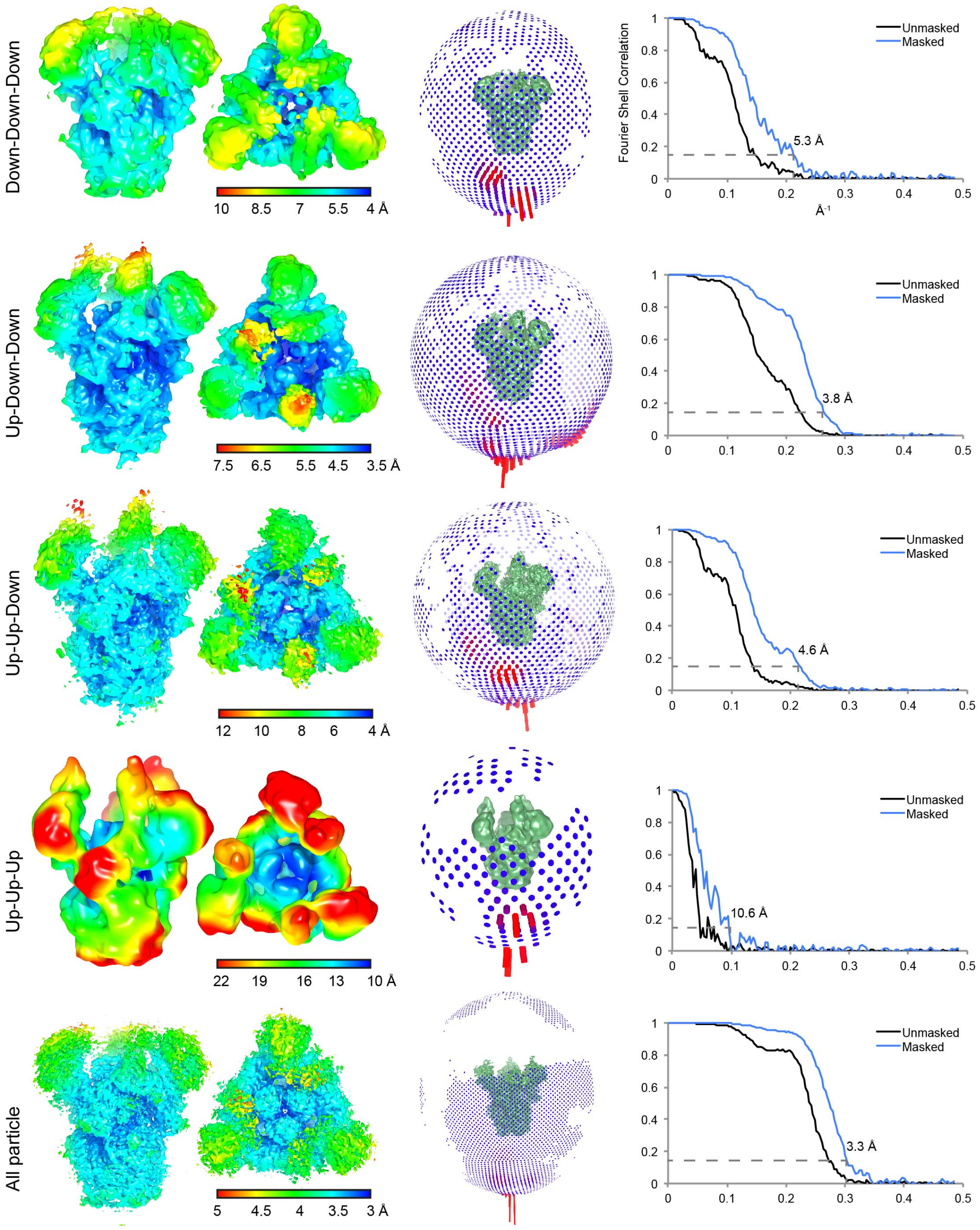

Supplemental Figure 10: Local Resolution, angular distribution and Fourier shell correlation plots for structures derived from the SARS S 2P – trypsin cleaved dataset. Local resolution of each reconstruction (side and membrane distal views) was calculated in RELION[4] (left). Angular distribution was taken from the relion_refine output (center). Fourier

shell correlation (FSC) curves were calculated by relion_postprocess with and without a low-pass filtered map that had been extended by three pixels with a six pixel Gaussian soft edge (right).

References

1 Adams, P. D. *et al.* PHENIX: a comprehensive Python-based system for macromolecular structure solution. *Acta crystallographica. Section D, Biological crystallography* **66**, 213-221, doi:10.1107/s0907444909052925 (2010).

2 Chen, V. B. *et al.* MolProbity: all-atom structure validation for macromolecular crystallography. *Acta crystallographica. Section D, Biological crystallography* **66**, 12-21, doi:10.1107/s0907444909042073 (2010).

3 Barad, B. A. *et al.* EMRinger: side chain-directed model and map validation for 3D cryo-electron microscopy. *Nature methods* **12**, 943-946, doi:10.1038/nmeth.3541 (2015).

4 Kimanius, D., Forsberg, B. O., Scheres, S. H. & Lindahl, E. Accelerated cryo-EM structure determination with parallelisation using GPUs in RELION-2. *eLife* **5**, doi:10.7554/eLife.18722 (2016).

5 Li, F., Li, W., Farzan, M. & Harrison, S. C. Structure of SARS coronavirus spike receptor-binding domain complexed with receptor. *Science (New York, N.Y.)* **309**, 1864-1868, doi:10.1126/science.1116480 (2005).
